# Uncalibrated Visual Servoing for Underwater Vehicle Manipulator Systems with an Eye in Hand Configuration Camera

**DOI:** 10.3390/s19245469

**Published:** 2019-12-11

**Authors:** Jiyong Li, Hai Huang, Yang Xu, Han Wu, Lei Wan

**Affiliations:** National Key Laboratory of Science and Technology of Underwater Vehicle, Harbin Engineering University, No.145 Nantong Street Harbin, Harbin 150001, Chinaxuyangnn@126.com (Y.X.); wuhan@hrbeu.edu.cn (H.W.); wanlei@hrbeu.edu.cn (L.W.)

**Keywords:** visual servoing, underwater vehicle manipulator system, uncalibrated camera, adaptive control, disturbance observer

## Abstract

This paper presents an uncalibrated visual servoing scheme for underwater vehicle manipulator systems (UVMSs) with an eye-in-hand camera under uncertainties. These uncertainties contain vision sensor parameters, UVMS kinematics and feature position information. At first, a linear separation approach is addressed to collect these uncertainties into vectors, and this approach can also be utilized in other free-floating based manipulator systems. Secondly, a novel nonlinear adaptive controller is proposed to achieve image error convergence by estimating these vectors, the gradient projection method is utilized to optimize the restoring moments. Thirdly, a high order disturbance observer is addressed to deal with time-varying disturbances, and the convergence of the image errors is proved under the Lyapunov theory. Finally, in order to illustrate the effectiveness of the proposed method, numerical simulations based on a 9 degrees of freedom (DOFs) UVMS with an eye-in-hand camera are conducted. In simulations, the UVMS is expected to track a circle trajectory on the image plane, meanwhile, time-varying disturbances are exerted on the system. The proposed scheme can achieve accurate and smooth tracking results during simulations.

## 1. Introduction

Nowadays, underwater vehicles play a pivotal role in the applications of oceanic explorations, submarine salvages and scientific expeditions [1,2]. Highly integrated underwater vehicles, e.g., Ocean One [3], SAUVIM I Intervention Autonomous Underwater Vehicle (I-AUV) [4], and Girona 500 I-AUV [5] are developed for the field intervention operations based on its camera-robot systems. Compared with remote operations of underwater vehicles, autonomous underwater operations by visual servoing not only reduce the dependence on the mother ship, but also decrease disturbances from the umbilical cables, which makes underwater vehicles complete manipulations autonomously with less operations from professional pilots [6,7].

Visual servoing based on the vision sensor (i.e., underwater camera) have gained significant attentions in underwater autonomous interventions. The purpose of visual servoing on underwater vehicles is to control the end-effector to complete the specific intervention tasks through visual information [8]. The visual servoing can be generously divided into two categories [9], the position-based visual servoing (PBVS) and the image-based visual servoing (IBVS). The PBVS scheme demands visual sensors to obtain three-dimensional (3 D) position of the target and then control the end-effector approach to the desired position, which means accurate calibration results of the camera-robot system are often indispensable [10]. Classical IBVS scheme guides the end-effector directly through the information of the target, its core skill is to calculate the inverse or the transpose of the image Jacobian matrix (interaction matrix) [11]. However, these mentioned schemes require intrinsic and extrinsic parameters of the camera. It is well known that camera calibrations are very costly and tedious, especially in underwater environment [12]. Therefore, in order to achieve satisfied visual servoing results in underwater interventions, it is not suitable to directly utilize either the PBVS scheme or the classical IBVS scheme.

To avoid the influences of inaccurate camera calibrations, without perfectly knowing actual parameters of the vision sensor, uncalibrated visual servoing based on the IBVS scheme has attracted great attentions in last twenty years. A large portion of works turned image Jacobian matrix calculation to a parameter identification problem by establishing proper cost functions [13]. Then, without knowing the exact knowledge of camera parameters in advance, the image Jacobian matrix in the IBVS scheme can be acquired through estimating its elements by numerical estimations (i.e., Broyden-based method [14], recursive least square method [15], Kalman method [16], SVM based method [17] etc.). However, such kinematic-based uncalibrated visual servoing schemes usually ignore the nonlinear forces in robot dynamics and assumed the controller of the system is ideal, further, they do not take control schemes into stability analysis.

Such drawbacks are largely remedied under dynamic based visual servoing schemes by using a depth-independent interaction matrix [18], this pioneering work proposed a method to deal with the case of time-varying and uncertain depth in visual servoing process. Wang et al. [19] investigated uncalibrated visual servoing problem with uncertain models, they presented the passivity properties in the overall kinematic system. In [20], Wang H. et al. proposed an adaptive controller with a velocity observer to avoid velocity measurement noises. For object grasping, a manipulator with an eye-in-hand camera is common, because the feature points should be extracted on the object, however, these articles only concern the situation about the eye-to-hand camera configuration. Liang et al. [21] proposed a unified method for both eye-in-hand and eye-to-hand camera configurations, this work also gave a separation algorithm on kinematic properties of the manipulator system, however, the uncertain kinematics of the manipulator was not considered. Wang et al. [22] proposed a dynamic uncalibrated visual servoing approach for a free-floating based manipulator, however, it assumed the system has no external disturbances, and this assumption is often hard to satisfy in underwater environment.

Although some researches that involve uncalibrated visual servoing have been launched on industrial manipulators and nonholonomic mobile robots, visual servoing for the UVMS is a largely under explored domain. Li et al. [23] presented a learning-based IBVS scheme for an absorptive underwater vehicle to realize autonomously sea-cucumbers capture. Gao et al. [24] proposed a hierarchical IBVS strategy for the underwater vehicle dynamic positioning problem. Sivčev et al. [25] proposed an engineering oriented PBVS scheme for work class hydraulic ROVs. The drawback with these strategies is, they require the accurate camera parameters in advance. In the underwater manipulation domain, there are some examples of researches on uncalibrated visual servoing, for example, Xu et al. [26] investigated a dynamic based uncalibrated visual servoing scheme for the underwater soft robot. However, to the best of our knowledge, there is limited work on dynamic based uncalibrated visual servoing concerned with the UVMS. Unlike industrial manipulators or mobile robots, the visual servoing process of the UVMS suffers from two aspects: on one hand, since the UVMS is a free-floating multibody mechanism, the inner coupling effects between the manipulator and the vehicle are strong and cannot be neglected [27]. On the other hand, the underwater environment is complex, which makes the system tend to be affected by currents, accurate calibration results of the camera-UVMS system are hard to achieve in underwater environment. These notable differences from other types of robots make it improper to directly employ previous visual servoing studies on UVMSs.

To solve these problems, we propose a novel uncalibrated visual servoing method for UVMSs with an eye-in-hand configuration camera. The main contributions of this work can be summarized as follows: firstly, we propose a novel linear separation method to collect some uncertainties into constant vectors compared with the one in [21,28]. This method can be applied for other free-floating based articulated manipulators. Secondly, to optimize the restoring moments of the UVMS, a new reference velocity term is proposed through exploiting the kinematic redundancy of the UVMS, moreover, without perfectly knowing the camera parameters, kinematics of the UVMS and the target position, a novel composite controller with new adaptive laws is proposed to solve the visual servoing problem. Thirdly, considering time-varying disturbances of the UVMSs, a high order disturbance observer is presented to estimate and compensate such disturbances, and stability analysis is given under the Lyapunov theory.

**Figure 1:**
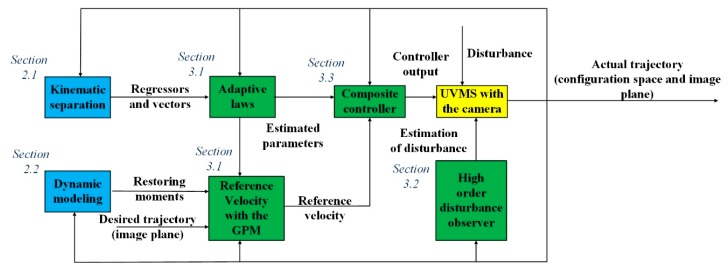
Flow chart of this work.

The rest of this paper is organized, as follows: in Section 2, we introduce the kinematic relationship of the system in UVMS, the dynamic model of the UVMS is also established. Thereafter, a novel adaptive controller with a disturbance observer is proposed for uncalibrated visual servoing in Section 3, stability proof analysis is also given is this section. In Section 4, numerical simulations on a 9-DOFs UVMS are carried out to demonstrate the effectiveness of the proposed scheme. The flow chart of this work is given in Figure 1.

Notation: Throughout this paper, we define $\varepsilon_{a,b}^{c}$ as the vector from the frame a to the frame b expressed in the frame c. $R_{a}^{b}$ and $T_{a}^{b}$ are the rotation matrix and the homogeneous transformation matrix from the frame a to the frame b respectively. Moreover, frames I,V,ee,fe,cam represent the inertial frame, the vehicle fixed frame, the end-effector frame, the target frame, and the camera frame respectively, frame i∈{0,1,2…n} represents the frame of the i-th joint.

## 2. Kinematics and Dynamics

In underwater visual servoing, camera parameters, UVMS kinematic modelling, and feature position are always difficult to accurately achieve. A linear separation method to collect camera uncertainties into vectors is given in [18,21], however this method can only be used for the fixed manipulator and under the assumption that kinematics of the manipulator is perfectly known. In this section, we propose a novel linear separation method to collect these uncertainties into constant vectors without this assumption for free-floating based articulated manipulators.

### 2.1. Kinematics

In this paper, we consider a 6+n DOFs UVMS with a standard fixed pinhole camera, the camera is fixed on the end-effector. A target is fixed w.r.t. to the inertial frame, and the feature point is extracted from the target. The image coordinates $x={\left[u,v\right]}^{T}$ of the feature point can be written as [18]:

(1) $$\left[\begin{array}{c} x\\ 1 \end{array}\right]=\frac{1}{z(\zeta )}{\mathrm{MT}}_{I}^{\mathrm{ee}}\left[\begin{array}{c} \varepsilon_{I,fe}^{I}\\ 1 \end{array}\right]$$

where z(ζ) is the depth of the feature in the camera frame. $M=\Phi T_{\mathrm{ee}}^{\mathrm{cam}}$, $M\in {\mathbb{R}}^{3\times 4}$ is the perspective projection matrix, which contains intrinsic and extrinsic parameters of the camera. $\Phi \in {\mathbb{R}}^{3\times 4}$ depends on intrinsic parameters of the camera and $T_{\mathrm{ee}}^{\mathrm{cam}}$ depends on extrinsic parameters of the camera. Since the camera is fixed on the end-effector, $T_{\mathrm{ee}}^{\mathrm{cam}}$ is time-invariant. Therefore, the perspective projection matrix M can be concluded to be a constant. Define ${M=[m}_{1}{,m}_{2}{,m}_{3}{]}^{T}$, $m_{i}\in {\mathbb{R}}^{1\times 4}$ is the *i*-th column of M. Depth z(ζ) can be obtained as:

(2) $$z(\zeta )=m_{3}^{T}T_{I}^{\mathrm{ee}}\left[\begin{array}{c} \varepsilon_{I,fe}^{I}\\ 1 \end{array}\right]$$

consider a UVMS with a n-DOFs manipulator, define $\dot{\zeta }{=[v}_{v}^{T},\omega_{v}^{T},{\dot{q}}^{T}{]}^{T}\in {\mathbb{R}}^{(6+n)\times 1}$ as the generalized velocity term of the UVMS, where $v_{v}^{}\in {\mathbb{R}}^{3\times 1}$ and $\omega_{v}^{}\in {\mathbb{R}}^{3\times 1}$ denote the linear and angular velocity of the vehicle in the vehicle fixed frame. Let ${\dot{\eta }}_{v}{=[v}_{v}^{T},\omega_{v}^{T}{]}^{T}$ denote the generalized velocity of the vehicle. $q\in {\mathbb{R}}^{n\times 1}$ is the vector represents joints position of the manipulator. By differentiating (1), the relationship of $\dot{\zeta }$ and $\dot{x}$ can be expressed as:

(3) $$\dot{x}=\frac{1}{z(\zeta )}J(\zeta ,x)\dot{\zeta }$$

where $J(\zeta ,x)=\left[\begin{array}{c} {\bar{m}}_{1}^{T}-u{\bar{m}}_{3}^{T}\\ {\bar{m}}_{2}^{T}-v{\bar{m}}_{3}^{T} \end{array}\right]\frac{\partial }{\partial \zeta }(R_{I}^{\mathrm{ee}}\varepsilon_{I,fe}^{I}+t)$ is the depth- independent Jacobian matrix, $R_{I}^{\mathrm{ee}}$ and t are the rotation and translation of $T_{I}^{\mathrm{ee}}$. And it can be further divided into two parts [20]:

(4) $$J(\zeta ,x)=J_{\mathrm{zin}}(\zeta )-xJ_{\mathrm{zd}}(\zeta )$$

On one hand, $J_{\mathrm{zin}}(\zeta )$ is so called depth-rate-independent Jacobian matrix, which maps the generalized velocity term $\dot{\zeta }$ to a plane parallel to the image plane. $J_{\mathrm{zin}}(\zeta )$ can be represented as:

(5) $$J_{\mathrm{zin}}(\zeta )=\left[\begin{array}{c} {\bar{m}}_{1}^{T}\\ {\bar{m}}_{2}^{T} \end{array}\right]\frac{\partial }{\partial \zeta }(R_{I}^{\mathrm{ee}}\varepsilon_{I,fe}^{I}+t)$$

${\bar{m}}_{i}$ is the first three elements of $m_{i}^{}$.

On the other hand, $J_{\mathrm{zd}}(\zeta )$ is so called depth-rate-dependent Jacobian matrix, which describes the relationship between $\dot{\zeta }$ and $\dot{z}(\zeta )$. $J_{\mathrm{zd}}(\zeta )$ and $\dot{z}(\zeta )$ can be represented as:

(6) $$J_{\mathrm{zd}}(\zeta )={\bar{m}}_{3}^{T}\frac{\partial }{\partial \zeta }(R_{I}^{\mathrm{ee}}\varepsilon_{I,fe}^{I}+t)$$

(7) $$\dot{z}(\zeta )={\bar{m}}_{3}^{T}\frac{\partial }{\partial \zeta }(R_{I}^{\mathrm{ee}}\varepsilon_{I,fe}^{I}+t)\dot{\zeta }=J_{\mathrm{zd}}(\zeta )\dot{\zeta }$$

The overall kinematics (2), (5), (7) share following properties.

**Property** **1.** *For a vector*$\eta \in {\mathbb{R}}^{(6+n)\times 1}$*,*$J_{\mathrm{zin}}(\zeta )\eta$*can be represented as the product of a regressor matrix*$Y_{\mathrm{zin}}(\zeta ,\eta )$*and a constant vector*$a_{\mathrm{zin}}$.

(8) $$J_{\mathrm{zin}}(\zeta )\eta =Y_{\mathrm{zin}}(\zeta ,\eta )a_{\mathrm{zin}}$$

**Proof.**  First, we will prove that

(9) $$\frac{\partial }{\partial \zeta }(R_{I}^{\mathrm{ee}}\varepsilon_{I,fe}^{I}+t)=\frac{\partial }{\partial \zeta }(\varepsilon_{fe,I}^{\mathrm{ee}}-\varepsilon_{\mathrm{ee},\;I}^{\mathrm{ee}})=-\frac{\partial }{\partial \zeta }(R_{I}^{\mathrm{ee}}\varepsilon_{I,\mathrm{ee}}^{I})\;=-(R_{I}^{\mathrm{ee}}\frac{\partial \varepsilon_{I,\mathrm{ee}}^{I}}{\partial \zeta }+ϒ\varepsilon_{I,\mathrm{ee}}^{I})=Y_{\alpha }(\zeta ,\eta )a_{\alpha }$$

where $ϒ=\left[\begin{array}{c} \frac{\partial (r_{11}+r_{21}+r_{31})}{\partial \zeta }\\ \frac{\partial (r_{12}+r_{22}+r_{32})}{\partial \zeta }\\ \frac{\partial (r_{13}+r_{23}+r_{33})}{\partial \zeta } \end{array}\right]$, and $r_{ij}$ is the (*i,j*) element of $R_{I}^{\mathrm{ee}}$.

On one side, it can be found that:

(10) $$\begin{array}{l} \frac{\partial \varepsilon_{I,\mathrm{ee}}^{I}}{\partial \zeta }\eta =[\begin{array}{ccc} R_{V}^{I} & -(S(R_{V}^{I}\varepsilon_{V0}^{V})+S(R_{0}^{I}\varepsilon_{0,\mathrm{ee}}^{0}))R_{V}^{I} & J_{pos,man} \end{array}]\eta \\ \;=R_{V}^{I}\eta_{1,3}+R_{V}^{I}S(\eta_{4,6})\varepsilon_{V0}^{V}+R_{V}^{I}S(\eta_{4,6})R_{0}^{V}\varepsilon_{0,\mathrm{ee}}^{0}\;+J_{pos,man}\eta_{7,6+n} \end{array}$$

where $\eta_{i,j}$ is the matrix composed from the element $\eta_{i}$ to $\eta_{j}$, i.e., $\eta_{i,j}={\left[\eta_{i},\eta_{i+1\ldots }\eta_{j}\right]}^{T}$. $J_{pos,man}$ is the manipulator Jacobian matrix mapping $\dot{q}$ to ${\dot{\varepsilon }}_{0,\mathrm{ee}}^{I}$.S(⋅) is a function translating a vector $a={\left[ax,ay,az\right]}^{T}$ to a skew symmetric matrix as:

$$S(a)=\left[\begin{array}{ccc} 0 & -az & ay\\ az & 0 & -ax\\ -ay & ax & 0 \end{array}\right]$$

Inspired by [29], $\varepsilon_{0,\mathrm{ee}}^{0}$ can be further divided as:

(11) $$\varepsilon_{0,\mathrm{ee}}^{0}{=[I}_{3},R_{1}^{0}\ldots ,R_{i}^{0}\ldots ,R_{n-1}^{0}]\cdot \mathrm{pp}\cdot a_{\mathrm{ee}}{=Y}_{\mathrm{ee}}(q)a_{\mathrm{ee}}$$

where $\mathrm{pp}=\mathrm{diag}\left\{{\cos(q}_{1}{),\sin(q}_{1}),1,\ldots {\cos(q}_{n}{),\sin(q}_{n}),1\right\}$ and $a_{\mathrm{ee}}={\left[a_{1},a_{1},d_{1}\ldots a_{n},a_{n},d_{n}\right]}^{T}$. $q_{i}$ is the position of the *i*-th joint, $a_{i},d_{i}$ are the link length and link offset associated with the i-th link. Besides,

(12) $$J_{\mathrm{pos},\mathrm{man}}\eta_{7,6+n}=R_{0}^{I}{[S(z}_{0})\eta_{7,6+n}{}_{\left(1\right)}{(D}_{n}{-D}_{0})\mathrm{pp}\ldots {S(z}_{n-1})\eta_{7,6+n}{}_{\left(n\right)}{(D}_{n}{-D}_{n-1})\mathrm{pp}]a_{\mathrm{ee}}{=Y}_{\mathrm{man}}(\eta ,q)a_{\mathrm{ee}}$$

where $D_{i}=\mathrm{diag}\left\{I_{3}^{1}\ldots I_{3}^{i}{,0}_{3\times 3}^{i+1}\ldots 0_{3\times 3}^{n}\right\}$ and $z_{i}$ is the unit vector of frame i expressed in frame 0 of the manipulator.

On the other side,

(13) $$\varepsilon_{I,\;\mathrm{ee}}^{I}=\varepsilon_{I,V}^{I}+R_{0}^{I}\varepsilon_{V,0}^{0}+R_{0}^{I}Y_{\mathrm{ee}}(q)a_{\mathrm{ee}}=Y_{\mathrm{eeb}}(q)a_{\mathrm{eeb}}$$

where $Y_{\mathrm{eeb}}(q)=[\varepsilon_{I,V}^{I},R_{0}^{I},R_{0}^{I}Y_{\mathrm{ee}}(q)]$, $a_{\mathrm{eeb}}={\left[I_{3\times 1},\varepsilon_{V,0}^{0},a_{\mathrm{ee}}^{T}\right]}^{T}$.

Therefore, by invoking (10)–(13), one can obtain:

$$Y_{\alpha }(\zeta ,\eta )=-[R_{V}^{\mathrm{ee}}\eta_{1,3},R_{V}^{\mathrm{ee}}S(\eta_{4,6})\varepsilon_{V0}^{V},R_{V}^{\mathrm{ee}}S(\eta_{4,6})R_{0}^{V}Y_{\mathrm{ee}}(q),R_{I}^{\mathrm{ee}}Y_{\mathrm{man}}(\eta ,q),ϒY_{\mathrm{eeb}}(q)]$$

$$a_{\alpha }={\left[\begin{array}{ccccc} I_{1\times 3} & I_{1\times 3} & a_{\mathrm{ee}}^{T} & a_{\mathrm{ee}}^{T} & a_{\mathrm{eeb}}^{T} \end{array}\right]}^{T}$$

and results can be further simplified as:

(14) $$Y_{\alpha }(\zeta ,\eta )=-[R_{V}^{\mathrm{ee}}\eta_{1,3}+R_{V}^{\mathrm{ee}}S(\eta_{4,6})r_{V0}^{V},R_{V}^{\mathrm{ee}}S(\eta_{4,6})R_{0}^{I}Y_{\mathrm{ee}}(q)\;+R_{I}^{\mathrm{ee}}Y_{\mathrm{man}}(\eta ,q),ϒY_{\mathrm{eeb}}(q)]$$

(15) $$a_{\alpha }=[\begin{array}{ccc} I_{1\times 3} & a_{\mathrm{ee}}^{T} & a_{\mathrm{eeb}}^{T} \end{array}{]}^{T}$$

To achieve the regressor matrix $Y_{\mathrm{zin}}(\zeta ,\eta )$, $Y_{\alpha }$ need to be transformed in to $Y_{\alpha s}\in {\mathbb{R}}^{1\times 3n}$ as:

$$Y_{\alpha s}(\zeta ,\eta ){=[y}_{\alpha 11},y_{\alpha 12},y_{\alpha 13},\ldots y_{\alpha n1},y_{\alpha n2},y_{\alpha n3}]$$

$y_{\alpha ij}$ is the (*i,j*) element of $Y_{\alpha }$.

Finally, from (5), (14), and (15), it can be obtained that:

(16) $$Y_{\mathrm{zin}}(\zeta ,\eta )=\left[\begin{array}{cc} Y_{\alpha s} & 0\\ 0 & Y_{\alpha s} \end{array}\right]$$

(17) $$a_{z\mathrm{in}}={\left[{\bar{m}}_{1}^{T}a_{\alpha 1}\ldots {\bar{m}}_{1}^{T}a_{\alpha n},{\bar{m}}_{2}^{T}a_{\alpha 1}\ldots {\bar{m}}_{2}^{T}a_{\alpha n}\right]}^{T}$$

where $a_{\alpha i}$ is the *i*-th element of the vector $a_{\alpha }$.□

**Property** **2.** *For a vector* $\phi \in {\mathbb{R}}^{2\times 1}$ *,* $\dot{z}(\zeta )\phi$ *can be represented as the product of a regressor matrix and a constant vector.*

(18) $$\dot{z}(\zeta )\phi =Y_{\mathrm{zd}}(\zeta ,\phi )a_{\mathrm{zd}}$$

**Proof.**  From (7) and the proof on the Property 1, it can be found that:

(19) $$Y_{\mathrm{zd}}=\left[\begin{array}{c} \phi_{1}Y_{\alpha s}(\zeta ,\dot{\zeta })\\ \phi_{2}Y_{\alpha s}(\zeta ,\dot{\zeta }) \end{array}\right]$$

(20) $$a_{\mathrm{zd}}={\left[{\bar{m}}_{3}^{T}a_{\alpha 1}\ldots {\bar{m}}_{3}^{T}a_{\alpha n}\right]}^{T}$$

where $\phi_{i}$ is the i-th element of the vector ϕ.□

**Property** **3.** *For a vector* $\phi \in {\mathbb{R}}^{2\times 1}$ *,* z(ζ)ϕ *can be represented as the product of a regressor matrix and a constant vector.*

(21) $$z(\zeta )\phi =Y_{z}(\zeta ,\phi )a_{z}$$

**Proof.**  According to (2), z(ζ)ϕ can be extended as:

(22) $$\begin{array}{l} z(\zeta )\phi =m_{3}^{T}T_{I}^{\mathrm{ee}}\left[\begin{array}{c} \varepsilon_{I,fe}^{I}\\ 1 \end{array}\right]\phi =(\sum_{i=1}^{3}r_{i1}m_{3i}^{}\varepsilon_{I,fe1}^{I}+\sum_{i=1}^{3}r_{i2}m_{3i}^{}\varepsilon_{I,fe2}^{I}\\ \;+\sum_{i=1}^{3}r_{i3}m_{3i}^{}\varepsilon_{I,fe3}^{I}+{\bar{m}}_{3}^{T}R_{I}^{\mathrm{ee}}\varepsilon_{\mathrm{ee},I}^{I}{+m}_{34}^{})\left[\begin{array}{c} \phi_{1}\\ \phi_{2} \end{array}\right] \end{array}$$

where $m_{3i}^{}$ and $\varepsilon_{I,fei}^{I}$ are the i-th elements of the vector $m_{3}^{}$ and the vector $\varepsilon_{I,fe}^{I}$, respectively. From (13), one obtains:

(23) $$Y_{z}(\zeta ,\phi )=\left[\begin{array}{ccc} \bar{R}\phi_{1} & \phi_{1}\sum_{j=1}^{3}\sum_{i=1}^{3}r_{ji}y_{\mathrm{eebj}} & \phi_{1}\\ \bar{R}\phi_{2} & \phi_{1}\sum_{j=1}^{3}\sum_{i=1}^{3}r_{ji}y_{\mathrm{eebj}} & \phi_{2} \end{array}\right]$$

(24) $$a_{z}=[{\bar{m}}_{3}^{T}\varepsilon_{I,fe1}^{I}{\bar{m}}_{3}^{T}\varepsilon_{I,fe2}^{I}{\bar{m}}_{3}^{T}\varepsilon_{I,fe3}^{I}\;-{(m}_{31}{+m}_{32}{+m}_{33})a_{\mathrm{eeb}}^{T}{\;m}_{34}{]}^{T}$$

where $\bar{R}{=[r}_{11}{\;r}_{21}{\;r}_{31}{\;r}_{12}{\;r}_{22}{\;r}_{32}{\;r}_{13}{\;r}_{23}{\;r}_{33}]$, $y_{\mathrm{eeb}}{}_{j}$ is the *j*-th row of the matrix $Y_{\mathrm{eeb}}(q)$.□

Therefore, $J_{\mathrm{zin}}(\zeta )\eta$, $\dot{z}(\zeta )\phi$, z(ζ)ϕ can be linearly separated as the product of the regressor matrices and the constant parameter vectors.

As described in previous proofs, camera perspective projection matrix M, the manipulator kinematics parameters $a_{\mathrm{ee}}$ and the manipulator relative position vector $\varepsilon_{V,0}^{0}$ are all collected in $a_{\mathrm{zin}}$,$a_{z}$,$a_{\mathrm{zd}}$. The position of the target $\varepsilon_{I,fe}^{I}$ is collected in $a_{z}$.

This work devotes to realize visual servoing under uncalibrated conditions. From the proofs of above three properties, uncertainties of the systems (i.e., uncertainties in intrinsic and extrinsic parameters of the camera, the kinematics of the UVMS, the position of the target w.r.t. the inertial frame) can be collected in these three parameter vectors in (17), (20), and (24). Because the target is fixed with the inertial frame, these vectors maintain constant and will be estimated by gradient update laws in the following section

By defining $\Delta a=a-a_{d}$, a compensated depth-Jacobian matrix $J_{\Omega }$ can be obtained by combining $J_{\mathrm{zin}}(\zeta )$ with $J_{\mathrm{zd}}(\zeta )$ [19,21].

(25) $$J_{\Omega }=J_{\mathrm{zin}}(\zeta )-\frac{x+x_{d}}{2}J_{\mathrm{zd}}(\zeta )=J(\zeta ,x)+\frac{\Delta x}{2}J_{\mathrm{zd}}(\zeta )$$

where $x_{d}$ is the bounded desired position of the feature point on the image plane. Moreover, ${\dot{x}}_{d}$ and ${\ddot{x}}_{d}$ are the bounded desired velocity and desired acceleration of the feature point, respectively.

Substituting (3) and (4) into (25), (3) can be rewritten as:

(26) $$J_{\Omega }\dot{\zeta }=z\dot{x}+\frac{1}{2}\dot{z}\Delta x\Rightarrow J_{\Omega }\dot{\zeta }-z{\dot{x}}_{d}=z\Delta \dot{x}+\frac{1}{2}\dot{z}\Delta x$$

$J_{\Omega }$ is used to describe the relationship from the generalized velocity term $\dot{\zeta }$ to $z\dot{x}+\frac{1}{2}\dot{z}\Delta x$.

### 2.2. Dynamics

**Figure 2:**
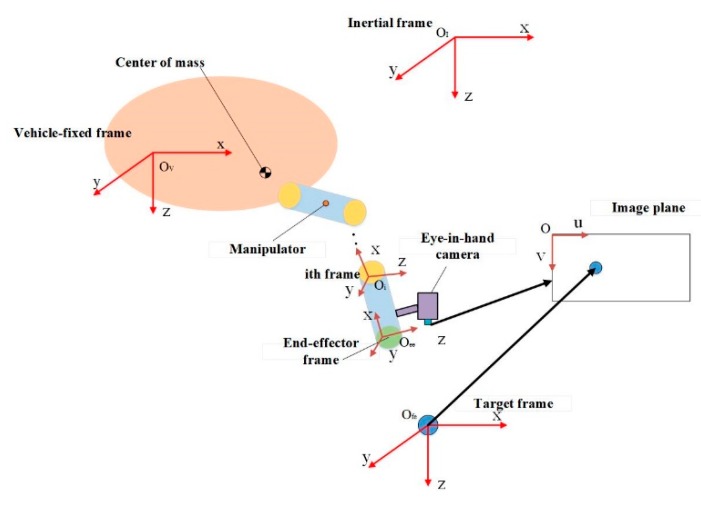
Coordinate frames of the UVMS.

Coordinate frames of a typical UVMS with an eye-in-hand configuration camera can be represented in Figure 2.

Let $\eta_{\mathrm{ee}}=[\varepsilon_{0,ee}^{I}{}^{T},\varphi_{ee},\theta_{ee},\psi_{ee}{]}^{T}$ denote the position and Euler angle vector of the end-effector. The relationship between ${\dot{\eta }}_{\mathrm{ee}}$ and $\dot{\zeta }$ can be expressed as:

(27) $${\dot{\eta }}_{\mathrm{ee}}=J_{\mathrm{uvms}}\dot{\zeta }$$

where $J_{\mathrm{uvms}}$ is the Jacobian matrix of the UVMS. Besides, the dynamic equation in the vehicle-joint space of UVMS can be written as [30]:

(28) $$M(\zeta )\ddot{\zeta }+C(\zeta ,\dot{\zeta })\dot{\zeta }+D(\zeta ,\dot{\zeta })\dot{\zeta }+G(\zeta )=\tau_{\mathrm{ctrl}}+\tau_{\mathrm{ext}}$$

$M(\zeta )\in {\mathbb{R}}^{(6+n)\times (6+n)}$ denotes the mass matrix including the added mass term, $C(\zeta ,\dot{\zeta })\in {\mathbb{R}}^{(6+n)\times (6+n)}$ represents the centrifugal and Coriolis effect matrix, $D(\zeta ,\dot{\zeta })\in {\mathbb{R}}^{(6+n)\times (6+n)}$ is the contribution due to the damping effects, $G(\zeta )\in {\mathbb{R}}^{(6+n)\times 1}$ represents the restoring matrix. $\tau_{\mathrm{ctrl}}\in {\mathbb{R}}^{(6+n)\times 1}$ is the controller output, $\tau_{\mathrm{ext}}\in {\mathbb{R}}^{(6+n)\times 1}$ represents the bounded unknown external disturbances on the UVMS, mainly caused by underwater currents, variation in load.

Specifically speaking, one can extend these dynamics matrices as:

$$M(\zeta )=\left[\begin{array}{cc} M_{v}(\eta_{v}) & M_{vm}(q)\\ M_{vm}^{T}(q) & M_{m}(q) \end{array}\right]$$

$$C(\zeta ,\dot{\zeta })=\left[\begin{array}{cc} C_{v}(\eta_{v},{\dot{\eta }}_{v}) & 0\\ 0 & C_{m}(q,\dot{q}) \end{array}\right]$$

$$D(\zeta ,\dot{\zeta })=\left[\begin{array}{cc} D_{v}(\eta_{v},{\dot{\eta }}_{v}) & 0\\ 0 & D_{m}(q,\dot{q}) \end{array}\right]$$

$$G(\zeta )=\left[\begin{array}{l} G_{v}(\eta_{v})\\ G_{m}(q) \end{array}\right]$$

Subscript v and m represent vehicle and manipulator, respectively. $M_{v}\in {\mathbb{R}}^{6\times 6}$ is the mass matrix of the sole vehicle, $M_{m}\in {\mathbb{R}}^{\mathrm{nxn}}$ is the mass matrix of the manipulator. $M_{\mathrm{vm}}\in {\mathbb{R}}^{6\mathrm{xn}}$ is the coupling term between the vehicle and the manipulator. Similar properties can also be found in $C(\zeta ,\dot{\zeta })$, $D(\zeta ,\dot{\zeta })$ and G(ζ). Comparing with traditional underwater vehicles and fixed manipulators, UVMSs are more complex due to multibody inner coupling effects.

Inspired by the inherent dynamic characteristics of UVMSs, $\hat{M}$ and $\hat{C}$ are chosen to satisfy following properties [30,31]:

**Property** **4.** *The inertial matrix* M(ζ) *is, positive and bounded:*

$$M(\zeta )>0\;\upsilon_{1}I\leq M(\zeta )\leq \upsilon_{2}I$$

$\upsilon_{1}$ *and* $\upsilon_{2}$ *are two positive constants.*

**Property** **5.** *The 2-norm of Coriolis matrix* $C(\zeta ,\dot{\zeta })$ *is bounded:*

$$\left\|C(\zeta ,\dot{\zeta })\right\|\leq \upsilon_{3}{\left\|\dot{\zeta }\right\|}_{\max}$$

*where* $\upsilon_{3}$ *is a positive scalar. The 2-norm means:*

$$x\in R^{n}\Rightarrow \left\|x\right\|=\sqrt{x^{T}x},\;x\in R^{\mathrm{nxn}}\Rightarrow \left\|x\right\|=\sqrt{\lambda_{\max}(x^{T}x})$$

*where* $\lambda_{\max}(\cdot )$ *is the maximum eigenvalue of a matrix.*

**Property** **6.** *The matrix* $\dot{M}(\zeta )-2C(\zeta ,\dot{\zeta })$ *is skew-symmetric:*

$$\dot{M}(\zeta )-2C(\zeta ,\dot{\zeta })=-{\left[\dot{M}(\zeta )-2C(\zeta ,\dot{\zeta })\right]}^{T}$$

From Properties 5 and 6, it can be found that:

$$\left\|\dot{M}(\zeta )\right\|\leq 2\upsilon_{3}{\left\|\dot{\zeta }\right\|}_{\max}$$

which means the 2-norm of $\dot{M}(\zeta )$ is also upper bounded.

Intrinsic dynamic parameters of a UVMS, e.g., the mass and the inertia tensor of each DOF, the buoyance of each DOF etc., are always difficult to accurately obtain. Hence, it is also difficult to acquire the accurate dynamic modeling in practice. Unmodeled dynamics, in addition to external disturbances are considered as system lumped disturbances. Then, the dynamic Equation (27) can be furthered represented as:

(29) $$\hat{M}(\zeta )\ddot{\zeta }+\hat{C}(\zeta ,\dot{\zeta })\dot{\zeta }+\hat{D}(\zeta ,\dot{\zeta })\dot{\zeta }+\hat{G}(\zeta )=\tau_{\mathrm{ctrl}}+\tau_{d}$$

where $\hat{M}(\zeta )$, $\hat{C}(\zeta ,\dot{\zeta })$, $\hat{D}(\zeta ,\dot{\zeta })$, $\hat{G}(\zeta )$ are nominal dynamic model matrices. $\tilde{M}(\zeta )$, $\tilde{C}(\zeta ,\dot{\zeta })$, $\tilde{D}(\zeta ,\dot{\zeta })$, $\tilde{G}(\zeta )$ are modeling error terms, and they are defined as: $\tilde{A}=A-\hat{A}$. $\tau_{d}=\tau_{\mathrm{ext}}-(\tilde{M}\ddot{\zeta }+\tilde{C}\dot{\zeta }+\tilde{D}\dot{\zeta }+\tilde{G})$ denotes the time varying disturbance term.

Restoring moments are caused by gravity and buoyancy forces. For the UVMS, the vehicle is easily affected by the restoring moments because of the movement of the manipulator. Therefore, it is important to decrease the restoring moments by choosing proper $\dot{\zeta }$. We assume that $\left\|\hat{G}(\zeta )\right\|$, $\left\|\frac{\partial \hat{G}(\zeta )}{\partial \zeta }\right\|$ and $\left\|\frac{\partial^{2}\hat{G}(\zeta )}{\partial \zeta^{2}}\right\|$ are all bounded.

**Remark** **1.** *Nominal dynamic model matrices* $\hat{M}(\zeta )$ *,* $\hat{C}(\zeta ,\dot{\zeta })$ *are chosen to satisfy Properties 4–6. In the following controller,* $\hat{M}(\zeta )$ *,* $\hat{C}(\zeta ,\dot{\zeta })$ *,* $\hat{D}(\zeta ,\dot{\zeta })$ *and* $\hat{G}(\zeta )$ *are obtained through choosing another set of intrinsic dynamic parameters (which is different from the actual intrinsic parameters, and it is utilized in forward dynamic routines in simulations), rather than updated by adaptive laws. In other word, we do not estimate or update nominal matrices, we directly calculate them by utilizing system states $\left[\zeta ,\dot{\zeta }\right]$ and intrinsic dynamic parameters. Therefore,* $\hat{M}(\zeta )$ *,* $\hat{C}(\zeta ,\dot{\zeta })$ *,* $\hat{D}(\zeta ,\dot{\zeta })$ *and* $\hat{G}(\zeta )$ *can be chosen to satisfy above properties.*

## 3. Controller Design

The control objective is to realize the convergence of image errors. In the process of designing the controller, at first, we establish a new reference velocity term to optimize the restoring moment through using the gradient projection method (GPM), moreover, novel adaptive laws are established to estimate the uncertainties in uncalibrated systems by utilizing the linear separation method which we described in the last section. Secondly, we employ a high-order disturbance observer to compensate the time-varying lumped disturbance term in (29). Thirdly, we propose a novel composite controller to guarantee the convergence of image errors, stability analysis based on the Lyapunov theory is also presented.

### 3.1. Adaptive Laws

To realize visual servoing, we should firstly design a reference velocity term in vehicle-joint space. If the kinematic parameters are available, then $a_{\mathrm{zin}}$,$a_{z}$,$a_{\mathrm{zd}}$ can be obtained. A reference velocity can be designed as:

(30) $${\dot{\zeta }}_{r}=J^{†}{}_{\Omega }z{\dot{x}}_{d}-\alpha_{v}J^{T}{}_{\Omega }z\Delta x$$

where $\alpha_{v}$ is a positive gain. Then, if there exists an ideal controller to make $\dot{\zeta }={\dot{\zeta }}_{r}$, substituting (30) into (26), one can find:

(31) $$-\alpha_{v}J_{\Omega }J^{T}{}_{\Omega }z\Delta x=z\Delta \dot{x}+\frac{1}{2}\dot{z}\Delta x$$

Consider a Lyapunov function as:

$$V_{\mathrm{temp}}=\frac{1}{2}z\Delta x^{T}\Delta x,$$

take the time-derivative of $V_{\mathrm{temp}}$, yields:

$${\dot{V}}_{\mathrm{temp}}=-\alpha_{v}\Delta x^{T}J_{\Omega }J^{T}{}_{\Omega }z\Delta x.$$

If $J_{\Omega }$ is non-singular, ${\dot{V}}_{\mathrm{temp}}<0$ and Δx→0 as t→∞.

To achieve tracking control, it is expected to acquire the accurate value of $J_{\Omega }$, z and $\dot{z}$, which equals to acquire the accurate value of $a_{\mathrm{zin}}$, $a_{z}$ and $a_{\mathrm{zd}}$. Unfortunately, it cannot be accurately obtained by considering the kinematic uncertainties of the system. Hence it is necessary to build adaptive laws to estimate them. The core is to estimate $a_{\mathrm{zin}}$, $a_{z}$ and $a_{\mathrm{zd}}$.

The compensated depth-Jacobian matrix in (25) can be estimated as:

(32) $${\hat{J}}_{\Omega }=\hat{J}(\zeta ,x)+\frac{\Delta x}{2}{\hat{J}}_{\mathrm{zd}}(\zeta )$$

By employing the pseudo inverse and transpose of ${\hat{J}}_{\Omega }$ in (32) and the estimation of depth $\hat{z}$, a reference velocity term that similar with the ones in [19,20,21] can be represented as:

(33) $${\dot{\zeta }}_{r}={\hat{J}}^{†}{}_{\Omega }\hat{z}{\dot{x}}_{d}-\alpha_{v}{\hat{J}}^{T}{}_{\Omega }\hat{z}\Delta x$$

where ${\hat{J}}^{†}{}_{\Omega }$ denotes the pseudo inverse of ${\hat{J}}_{\Omega }$, $\hat{z}$ is the estimated depth.

The UVMS possesses relatively high DOFs compared with simple manipulators in [19,20,21], hence the redundancy of the UVMS should be taken into consideration when design the reference velocity. Because the UVMS is easily be affected by the restoring moment, the GPM is utilized to optimize the restoring moments through exploiting kinematic redundancy, a novel reference velocity can be represented as:

(34) $${\dot{\zeta }}_{r}={\hat{J}}^{†}{}_{\Omega }\hat{z}{\dot{x}}_{d}-\alpha_{v}{\hat{J}}^{T}{}_{\Omega }\hat{z}\Delta x-\kappa_{v}(I-{\hat{J}}^{†}{}_{\Omega }{\hat{J}}^{}{}_{\Omega })\nabla P_{r}$$

where $\kappa_{v}$ is a positive gain. $\nabla P_{r}$ is defined as $\nabla P_{r}={\hat{G}}^{T}(\zeta )W_{G}^{}\frac{\partial \hat{G}(\zeta )}{\partial \zeta }$, $W_{G}^{}$ is a positive weight matrix. $\left(I-{\hat{J}}^{†}{}_{\Omega }{\hat{J}}^{}{}_{\Omega }\right)$ projects $\nabla P_{r}$ into the null space of ${\hat{J}}^{}{}_{\Omega }$, hence $\nabla P_{r}$ is only effective in the null space.

Although some previous works utilize the GPM to optimize restoring moments for UVMSs [32,33,34], there are two main differences. One is the Jacobian matrix in [32,33,34] is used to describe the velocity relationship between the configuration space and the Cartesian space (i.e., the Jacobian matrix in (27)), rather than the Jacobian matrix described in (26). The other one is previous works [32,33,34] all depend on the accurate Jacobian matrix, rather than the estimated Jacobian matrix.

Then, a sliding vector can be defined as:

(35) $$s=\dot{\zeta }-{\dot{\zeta }}_{r}$$

estimation error terms can be defined as: $\Delta a_{\mathrm{zin}}^{}={\hat{a}}_{\mathrm{zin}}^{}-a_{\mathrm{zin}}^{}$, $\Delta a_{\mathrm{zd}}^{}={\hat{a}}_{\mathrm{zd}}^{}-a_{\mathrm{zd}}^{}$, $\Delta a_{z}^{}={\hat{a}}_{z}^{}-a_{z}^{}$. Then, substituting (7), (18) into (25), one can find:

(36) $$({\hat{J}}_{\Omega }-J_{\Omega })\dot{\zeta }=({\hat{J}}_{\mathrm{zin}}(\zeta )-J_{\mathrm{zin}}(\zeta ))\dot{\zeta }-\frac{x+x_{d}}{2}({\hat{J}}_{\mathrm{zd}}(\zeta )-J_{\mathrm{zd}}(\zeta ))\dot{\zeta }=Y_{\mathrm{zin}}(\zeta ,\dot{\zeta })\Delta a_{\mathrm{zin}}-\frac{1}{2}Y_{\mathrm{zd}}(\zeta ,\dot{\zeta },x+x_{d})\Delta a_{\mathrm{zd}}$$

From (21) and (34), one has:

(37) $${\hat{J}}_{\Omega }{\dot{\zeta }}_{r}=\hat{z}{\dot{x}}_{d}-\alpha_{v}\hat{z}{\hat{J}}_{\Omega }{\hat{J}}_{\Omega }^{T}\Delta x=Y_{z}(\zeta ,{\dot{x}}_{d}-\alpha_{v}{\hat{J}}_{\Omega }{\hat{J}}_{\Omega }^{T}\Delta x)\Delta a_{z}$$

Invoking (26), (34)–(36), the closed-loop kinematics system can be represented as:

(38) $$z\Delta \dot{x}+\frac{1}{2}\dot{z}\Delta x=Y_{z}(\zeta ,{\dot{x}}_{d}-\alpha_{v}{\hat{J}}_{\Omega }{\hat{J}}_{\Omega }^{T}\Delta x)\Delta a_{z}+{\hat{J}}_{\Omega }s+Y_{zd}(\zeta ,\dot{\zeta },\frac{x+x_{d}}{2})\Delta a_{zd}-Y_{zin}(\zeta ,\dot{\zeta })\Delta a_{zin}-\alpha_{v}z{\hat{J}}_{\Omega }{\hat{J}}_{\Omega }^{T}\Delta x$$

To calculate the reference velocity term in (34) and guarantee the stability of the system, it is necessary to estimate the unknown parameters of the systems (i.e., the constant vectors in (8), (18) and (21)). The adaptive update laws are designed as:

(39) $${\dot{\hat{a}}}_{\mathrm{zin}}=\lambda_{\mathrm{zin}}Y_{\mathrm{zin}}^{T}(\zeta ,\dot{\zeta })\Delta x$$

(40) $${\dot{\hat{a}}}_{\mathrm{zd}}=-\lambda_{\mathrm{zd}}Y_{\mathrm{zd}}^{T}(\zeta ,\dot{\zeta },\frac{x+x_{d}}{2})\Delta x$$

(41) $${\dot{\hat{a}}}_{z}=-\lambda_{z}Y_{z}^{T}(\zeta ,{\dot{x}}_{d}-\alpha {\hat{J}}_{\Omega }{\hat{J}}^{T}{}_{\Omega }\Delta x)\Delta x$$

$\lambda_{\mathrm{zin}}$, $\lambda_{\mathrm{zd}}$ and $\lambda_{z}$ are the positive gain matrices, respectively.

The novel reference velocity term (34), update laws (39)–(41) and stability analysis in the end of this section is one of the main contributions of this work.

**Remark** **2.** *We assume that the update laws (39) and (40) will not lead to the singularity of* ${\hat{J}}_{\Omega }$ *. In other words,* ${\hat{J}}_{\Omega }$ *is supposed to be non-singular. This is a common assumption in uncalibrated visual servoing works [19,20,21]. Besides, one may adopt the strategy presented in [35] to avoid the singular on* ${\hat{J}}_{\Omega }$ *.*

### 3.2. High Order Disturbance Observer

When compared with nonlinear disturbance observer in [31,36], the performance under high order disturbance observer (HODO) is better, because the disturbance model refers to high order information of the disturbance. Besides, in order to prove the asymptotic stability of the system, the disturbance term is often supposed to be constant in previous works [31,36]. This assumption can be cancelled under the high order disturbance observer.

The purpose of employing a disturbance observer is to estimate and compensate the time-varying unknown lumped disturbance term $\tau_{d}$. The philosophy of designing the disturbance observer is to estimate the unmeasurable term $\tau_{d}$ through measurable states. To establish the high order disturbance observer, we first rewrite the closed loop system (29) as a form as state space:

(42) $$\left\{ \begin{array}{l} {\dot{x}}_{1}=x_{2}\\ {\dot{x}}_{2}=\underset{f}{\underset{︸}{{\hat{M}}^{-1}(\tau_{\mathrm{ctrl}}-\hat{C}x_{2}-\hat{D}x_{2}-\hat{G})}}+\underset{d}{\underset{︸}{{\hat{M}}^{-1}\tau_{d}}} \end{array}\right.$$

where $x_{1}=\zeta$, $x_{2}=\dot{\zeta }$ denote the system variable states. Besides, $f={\hat{M}}^{-1}(\tau_{\mathrm{ctrl}}-\hat{C}x_{2}-\hat{D}x_{2}-\hat{G})$ is available in the system, $d={\hat{M}}^{-1}\tau_{d}$ is the unknown disturbance term to be estimated.

Suppose the disturbance term d has a bounded $r^{th}$ derivative,

$$\left|d^{\left(r\right)}\right|\leq \delta_{0}$$

where $\delta_{0}$ is the upper bound. The disturbance term d can be described through a linear system, given by [37]

(43) $$\left\{ \begin{array}{l} \dot{\xi }=W\xi +Ed^{\left(r\right)}\\ d=L\xi \end{array}\right.$$

where $\xi \in {\mathbb{R}}^{(6+n)\cdot r}$ is an auxiliary variable. $W\in {\mathbb{R}}^{(6+n)\cdot r\times (6+n)\cdot r}$, $E\in {\mathbb{R}}^{\left(6+n)\cdot r\times (6+n\right)}$, and $L\in {\mathbb{R}}^{(6+n)\times (6+n)\cdot r}$ can be represented as the following forms:

$$W=\left[\begin{array}{cc} 0_{\left(6+n)\cdot (r-1)\times (6+n\right)} & I_{(6+n)\cdot (r-1)}\\ 0_{\left(6+n)\times (6+n\right)} & 0_{\left(6+n)\times (6+n)\cdot (r-1\right)} \end{array}\right]$$

$$E=\left[\begin{array}{c} 0_{\left(6+n)\cdot (r-1)\times (6+n\right)}\\ I_{6+n} \end{array}\right]$$

$$L=[\begin{array}{cc} I_{6+n} & 0_{\left(6+n)\times (6+n)\cdot (r-1\right)} \end{array}]$$

Combining (42) and (43), one can obtains:

(44) $$\left\{ \begin{array}{l} \dot{\xi }=W\xi +ED^{\left(r\right)}\\ {\dot{x}}_{2}=f+L\xi \end{array}\right.$$

Then a new extended system can be derived through introducing an auxiliary variable $\gamma ={\left[\begin{array}{cc} \gamma_{1} & \gamma_{2} \end{array}\right]}^{T}$ as follows:

(45) $$\left[\begin{array}{c} \gamma_{1}\\ \gamma_{2} \end{array}\right]=\left[\begin{array}{c} x_{2}\\ -p(x_{2})+\xi \end{array}\right]$$

where $p(x_{2})$ is a polynomial vector to be designed. Differentiating (45) with respect to time and substituting (44), the dynamics of γ can be represented as:

(46) $$\left\{ \begin{array}{l} {\dot{\gamma }}_{1}=f+L(p(\gamma_{1})+\gamma_{2})\\ {\dot{\gamma }}_{2}=(W-l(\gamma_{1})L)\gamma_{2}+T+ED^{\left(r\right)} \end{array}\right.$$

where $l(\gamma_{1})=\frac{\partial p(\gamma_{1})}{\partial \gamma_{1}}$ is a vector to be designed. $T=Wp(\gamma_{1})-l(\gamma_{1})(f+Lp(\gamma_{1}))$, it is available through system measurable states and pre-designed vector $l(\gamma_{1})$.

Then, a high order disturbance observer can be derived as:

(47) $$\left\{ \begin{array}{l} {\dot{\hat{\gamma }}}_{2}=(W-l(\gamma_{1})L){\hat{\gamma }}_{2}+T\\ \hat{\xi }={\hat{\gamma }}_{2}+p(\gamma_{1})\\ \hat{d}=L\hat{\xi } \end{array}\right.$$

Finally, the estimation of $\tau_{d}$ follows:

(48) $${\hat{\tau }}_{d}=\hat{M}\hat{d}$$

The estimation error is defined as $e_{\xi }=\xi -\hat{\xi }$. And it is governed by following dynamic system:

(49) $${\dot{e}}_{\xi }=\dot{\xi }-\dot{\hat{\xi }}=W\xi +Ed^{\left(r\right)}-(W-l(\gamma_{1})L){\hat{\gamma }}_{2}-T-\dot{p}(\gamma_{1})\;=(W-l(\gamma_{1})L)e_{\xi }+Ed^{\left(r\right)}$$

The principle of designing $l(\gamma_{1})$ is to make the dominant pole of (49) lie in the left-half plane, then the system described in (49) is bounded-input bounded-output (BIBO) stable. If $l(\gamma_{1})$ is available, then $p(\gamma_{1})$ can be obtained from the relationship $l(\gamma_{1})=\frac{\partial p(\gamma_{1})}{\partial \gamma_{1}}$. Since the system is BIBO stable, the disturbance estimation error is bounded and satisfies:

$$\left\|d-\hat{d}\right\|\leq \delta_{1}$$

where $\delta_{1}$ is the upper bound. Since $\hat{M}$ is bounded, it can be deduced that:

$$\left\|\tau_{d}-{\hat{\tau }}_{d}\right\|=\left\|\hat{M}(d-\hat{d})\right\|\leq \left\|\hat{M}\right\|\left\|\left(d-\hat{d}\right)\right\|\leq \sigma$$

where σ is an unknown bounded constant.

### 3.3. Composite Controller

Now, by using the reference velocity ${\dot{\zeta }}_{r}$, the sliding vector s, the image error Δx, the disturbance estimation ${\hat{\tau }}_{d}$ and the approximation of unknown bound $\hat{\sigma }$, a composite controller with the HODO for uncalibrated visual servoing can be designed as:

(50) $$\tau_{\mathrm{ctrl}}=\hat{M}({\ddot{\zeta }}_{r}-K_{p}s)-K_{s}{\hat{J}}^{T}{}_{\Omega }\Delta x+\hat{C}{\dot{\zeta }}_{r}+\hat{D}\dot{\zeta }+\hat{G}\;-{\hat{\tau }}_{d}-\hat{\sigma }\mathrm{sgn}(s)$$

where $K_{p}$ and $K_{s}$ are positive diagonal matrices. In the controller, the first two terms are the feedback control term in vehicle-joint space and image space, receptively. $\hat{C}{\dot{\zeta }}_{r}+\hat{D}\dot{\zeta }+\hat{G}$ is the feed-forward control term. In vehicle-joint space, the reference velocity term ${\dot{\zeta }}_{r}$ is provided by (34), (39)–(41) through estimating $J_{\Omega }$ and z. ${\hat{\tau }}_{d}$ is the estimation term from the disturbance observer in (48). $\hat{\sigma }$ is the estimated value of the constant bound σ, and it is updated as the following adaptive law:

(51) $$\dot{\hat{\sigma }}=\lambda_{\sigma }\left\|s\right\|$$

Substituting (50) into (29), The close-loop dynamics equation of the UVMS can be represented as:

(52) $$\hat{M}\dot{s}=-\hat{M}K_{p}s-\hat{C}s+\hat{M}\tilde{d}-K_{s}{\hat{J}}^{T}{}_{\Omega }\Delta x-\hat{\sigma }\mathrm{sgn}(s)$$

**Figure 3:**
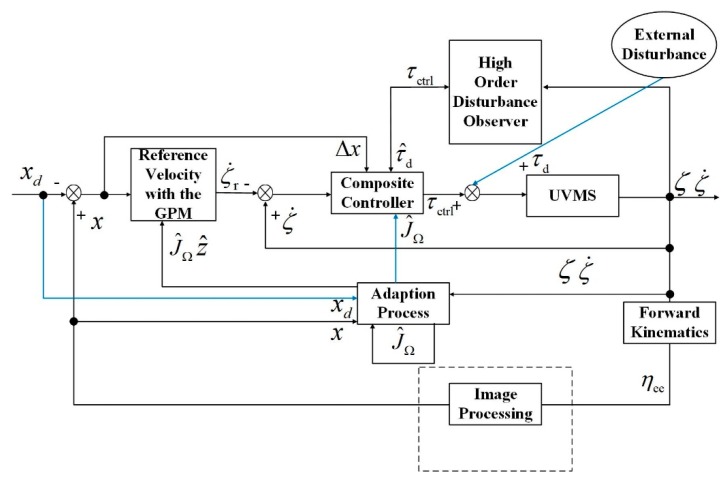
The architecture of the proposed scheme.

The whole architecture of the proposed scheme can be found in Figure 3.

**Theorem** **1.** *The adaptive laws (39)–(41), the high order disturbance observer (48), and the composite controller (50) guarantee the convergence of the image space tracking errors, i.e.,* Δx→0 *,* $\Delta \dot{x}\to 0$ *as* t→∞ *.*

**Proof.**  Consider a Lyapunov candidate function as:

(53) $$\begin{array}{l} V=\frac{1}{2}(z\Delta x^{T}\Delta x+\Delta a_{\mathrm{zin}}^{T}\lambda_{\mathrm{zin}}^{-1}\Delta a_{\mathrm{zin}}^{}+\Delta a_{\mathrm{zd}}^{T}\lambda_{\mathrm{zd}}^{-1}\Delta a_{\mathrm{zd}}^{}+\Delta a_{z}^{T}\lambda_{z}^{-1}\Delta a_{z}^{}\\ \;+s^{T}K_{s}^{-1}\hat{M}s+\frac{\left\|K_{s}^{-1}\right\|}{\lambda_{\sigma }}{\tilde{\sigma }}^{2}) \end{array}$$

From Properties 1–3, unknown parameter vectors expressed in (8), (18) and (21) are all constant. By differentiating (53) w.r.t. time, one has

(54) $$\begin{array}{l} \dot{V}=\frac{1}{2}\Delta x^{T}\dot{z}\Delta x+\Delta x^{T}z\Delta \dot{x}+\Delta a_{\mathrm{zin}}^{T}\lambda_{\mathrm{zin}}^{-1}{\dot{\hat{a}}}_{\mathrm{zin}}^{}+\Delta a_{\mathrm{zd}}^{T}\lambda_{\mathrm{zd}}^{-1}{\dot{\hat{a}}}_{\mathrm{zd}}^{}+\Delta a_{z}^{T}\lambda_{z}^{-1}{\dot{\hat{a}}}_{z}^{}\\ \;+s^{T}K_{s}^{-1}\hat{M}\dot{s}+\frac{1}{2}s^{T}K_{s}^{-1}\dot{\hat{M}}s-\frac{\left\|K_{s}^{-1}\right\|}{\lambda_{\sigma }}\dot{\hat{\sigma }}\tilde{\sigma } \end{array}$$

take Property 6, (38)–(41), (50) into (54), one has:

(55) $$\begin{array}{l} \dot{V}=-\alpha_{v}z\Delta x^{T}{\hat{J}}_{\Omega }{\hat{J}}_{\Omega }^{T}\Delta x+\Delta x^{T}{\hat{J}}_{\Omega }^{}s-s^{T}K_{s}^{-1}\hat{M}K_{p}s+s^{T}K_{s}^{-1}\hat{M}\tilde{d}-s^{T}{\hat{J}}_{\Omega }^{T}\Delta x\\ \;-\hat{\sigma }s^{T}K_{s}^{-1}\mathrm{sgn}(s)-\frac{\left\|K_{s}^{-1}\right\|}{\lambda_{\sigma }}\dot{\hat{\sigma }}\tilde{\sigma }\\ \;=-\alpha_{v}z\Delta x^{T}{\hat{J}}_{\Omega }{\hat{J}}_{\Omega }^{T}\Delta x-s^{T}\hat{M}K_{p}s+s^{T}K_{s}^{-1}\hat{M}\tilde{d}-\hat{\sigma }s^{T}K_{s}^{-1}\mathrm{sgn}(s)-\tilde{\sigma }\left\|K_{s}^{-1}\right\|\left\|s\right\| \end{array}$$

Let $s^{T}K_{s}^{-1}=\eta_{s}$, since $K_{s}$ is a positive diagonal matrix, it can be found:

$${\mathrm{sgn}(s}^{T})=\mathrm{sgn}(\eta_{s})$$

since ${\hat{J}}_{\Omega }$ is non-singular and $\hat{M}$ is positive, one can find a nonnegative definite continuous function W(ζ) satisfies:

(56) $$\begin{array}{l} \dot{V}=-\alpha_{v}z\Delta x^{T}{\hat{J}}_{\Omega }{\hat{J}}_{\Omega }^{T}\Delta x-s^{T}K_{s}^{-1}\hat{M}K_{p}s+\eta_{s}\hat{M}\tilde{d}-\hat{\sigma }\eta_{s}\mathrm{sgn}(\eta_{s}^{T})-\tilde{\sigma }\left\|K_{s}^{-1}\right\|\left\|s\right\|\\ \;\leq -\alpha_{v}z\Delta x^{T}{\hat{J}}_{\Omega }{\hat{J}}_{\Omega }^{T}\Delta x-s^{T}\hat{M}K_{p}s+\sigma \left\|\eta_{s}\right\|-\hat{\sigma }\left\|\eta_{s}\right\|-\tilde{\sigma }\left\|\eta_{s}\right\|\\ \;=-\alpha_{v}z\Delta x^{T}{\hat{J}}_{\Omega }{\hat{J}}_{\Omega }^{T}\Delta x-s^{T}\hat{M}K_{p}s\\ \;=-W(\zeta )\leq 0 \end{array}$$

Integrate W(ζ) from time 0 to current time T, it can be obtained

(57) $$\int_{0}^{T}W(\zeta (r))dr\leq V(\zeta (0))-V(\zeta (T))\leq V(\zeta (0))$$

V(ζ(0)) is fixed by the choice of initial condition, therefore $\int_{0}^{T}W(\zeta (r))dr$ is bounded.

Since ${\hat{J}}_{\Omega }{\hat{J}}_{\Omega }^{T}$ is positive definite, from (56), $\dot{V}$ is negative semi-definite, which implies that Δx,$\Delta a_{\mathrm{zin}}^{}$,$\Delta a_{\mathrm{zd}}^{}$, $\Delta a_{z}^{}$,s and $\tilde{\sigma }$ are all bounded. Therefore, ${\hat{a}}_{\mathrm{zin}}$, ${\hat{a}}_{\mathrm{zd}}$, ${\hat{a}}_{z}$ and $\hat{\sigma }$ are all bounded. Then the boundness of ${\hat{J}}_{\mathrm{zin}}(\zeta )$, $\dot{\hat{z}}$, ${\hat{J}}_{z}(\zeta )$ and $\hat{z}$ can be obtained. Hence, the boundness ${\hat{J}}_{\Omega }$ can be concluded from (32). Since ${\dot{x}}_{d}$ and $x_{d}$ are all bounded, ${\dot{\zeta }}_{r}$ is also bounded. Because s and ${\dot{\zeta }}_{r}$ are all bounded, the boundness of $\dot{\zeta }$ can be achieved. From (3) and (7), it can be obtained that $\dot{x}$ and $\dot{z}$ are all bounded, hence the boundness of $\Delta \dot{x}$ can be achieved. From (39)–(41), the boundness of ${\dot{\hat{a}}}_{\mathrm{zin}}$,${\dot{\hat{a}}}_{\mathrm{zd}}$ and ${\dot{\hat{a}}}_{z}$ can be obtained. Therefore, the boundness of ${\dot{\hat{J}}}_{z}(\zeta )$ and ${\dot{\hat{J}}}_{\mathrm{zin}}(\zeta )$ can be achieved by differentiating (7) and (8), respectively. As ${\dot{\hat{J}}}_{\mathrm{zin}}(\zeta )-\frac{\dot{x}+{\dot{x}}_{d}}{2}{\hat{J}}_{z}(\zeta )-\frac{x+x_{d}}{2}{\dot{\hat{J}}}_{z}(\zeta )={\dot{\hat{J}}}_{\Omega }$, the boundness of ${\dot{\hat{J}}}_{\Omega }$ can also be achieved.

Differentiating (34) w.r.t. time, one has:

(58) $$\begin{array}{l} {\ddot{\zeta }}_{r}={\dot{\hat{J}}}^{†}{}_{\Omega }\hat{z}{\dot{x}}_{d}+{\hat{J}}^{†}{}_{\Omega }\dot{\hat{z}}{\dot{x}}_{d}+{\hat{J}}^{†}{}_{\Omega }\hat{z}{\ddot{x}}_{d}-\alpha {\dot{\hat{J}}}^{T}{}_{\Omega }\hat{z}\Delta x-\alpha_{v}{\hat{J}}^{T}{}_{\Omega }\dot{\hat{z}}\Delta x-\alpha_{v}{\hat{J}}^{T}{}_{\Omega }\hat{z}\Delta \dot{x}\\ \;+\kappa_{v}({\dot{\hat{J}}}^{†}{}_{\Omega }{\hat{J}}^{}{}_{\Omega }+{\hat{J}}^{†}{}_{\Omega }{\dot{\hat{J}}}^{}{}_{\Omega })\nabla P_{r}-\kappa_{v}(I-{\hat{J}}^{†}{}_{\Omega }{\hat{J}}^{}{}_{\Omega })\nabla^{2}P_{r}\dot{\zeta } \end{array}$$

because ${\dot{\hat{J}}}_{\Omega }$, $\dot{\hat{z}}$, ${\dot{x}}_{d}$, ${\ddot{x}}_{d}$, $\Delta \dot{x}$, $\dot{\zeta }$,$\left\|\hat{G}(\zeta )\right\|$, $\left\|\frac{\partial \hat{G}(\zeta )}{\partial \zeta }\right\|$ and $\left\|\frac{\partial^{2}\hat{G}(\zeta )}{\partial \zeta^{2}}\right\|$ are all bounded, the boundness of ${\ddot{\zeta }}_{r}$ and $\dot{s}$ can be achieved. Differentiating W w.r.t. time, it satisfies:

(59) $$\begin{array}{l} \dot{W}=\alpha_{v}\dot{z}\Delta x^{T}{\hat{J}}_{\Omega }{\hat{J}}_{\Omega }^{T}\Delta x+2\alpha_{v}z\Delta x^{T}{\hat{J}}_{\Omega }{\hat{J}}_{\Omega }^{T}\Delta \dot{x}+2\alpha_{v}z\Delta x^{T}{\dot{\hat{J}}}_{\Omega }{\hat{J}}_{\Omega }^{T}\Delta x\\ \;+2s^{T}K_{s}^{-1}\hat{M}K_{p}\dot{s}+s^{T}K_{s}^{-1}\dot{\hat{M}}K_{p}s \end{array}$$

$\dot{W}$ is bounded due to Properties 4 and 6. The boundness of (59) illustrates that W is uniformly continuous [38]. Consider the boundness of $\int_{0}^{t}W(\zeta (r))dr$, Barbalat’s lemma can be applied. Therefore, W→0 as t→∞, which means Δx→0,s→0 as t→∞.

By differentiating (3), it is clear that $\ddot{x}$ is bounded. Hence $\Delta \ddot{x}$ is bounded too, and $\Delta \dot{x}$ is uniformly continuous. Since the boundness of Δx is achieved, it can be concluded $\Delta \dot{x}\to 0$ as t→∞ by applying Barbalat’s lemma. □

## 4. Performance Analysis

**Figure 4:**
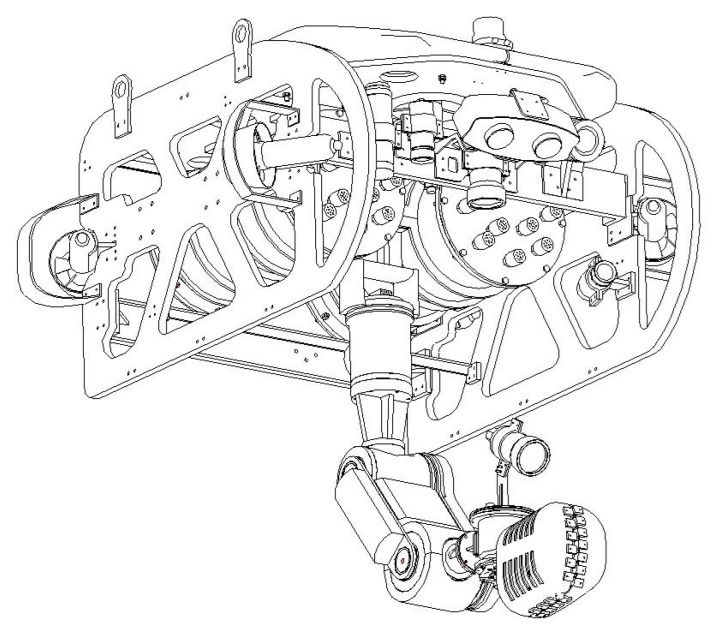
The configuration of the employed UVMS.

In order to demonstrate the effectiveness of the proposed controller, numerical simulations have been performed on a UVMS platform. As shown in Figure 4, the UVMS carries a 3 DOFs manipulator and the whole system contains 9 DOFs.

The dynamic parameters of the UVMS are displayed Table 1. The Denavit–Hartenberg table of the manipulator is showed in Table 2.

In simulations, an eye-in-hand camera with parameters $\alpha_{u}=50$ pixels, $\alpha_{v}=50$ pixels, $u_{0}=200$ pixels, $v_{0}=100$ pixels is employed. Parameters $\varepsilon_{\mathrm{cam},\mathrm{ee}}^{\mathrm{cam}}={\left(0.1,0.2,0.1\right)}^{T}$ m and $\varepsilon_{V,0}^{V}={\left(0,0,1.5\right)}^{T}$ m are employed to describe the kinematic relationships. The configuration of the manipulator follows Table 2. The target is fixed at the point $\varepsilon_{I,\mathrm{fe}}^{I}={\left(2.5,-6,5\right)}^{T}$ m w.r.t. the inertial frame.

We set initial values as follows: ${\hat{\alpha }}_{u}(0)=35$ pixels, ${\hat{\alpha }}_{v}(0)=75$ pixels, ${\hat{\alpha }}_{0}(0)=170$ pixels, ${\hat{\alpha }}_{0}(0)=210$ pixels, ${\hat{\varepsilon }}_{\mathrm{cam},\mathrm{ee}}^{\mathrm{cam}}(0)={\left(0.2,0.2,0.2\right)}^{T}$ m, ${\hat{\varepsilon }}_{V,0}^{V}(0)={\left(0.1,1.4,1\right)}^{T}$ m, ${\hat{\varepsilon }}_{I,\mathrm{fe}}^{I}(0)={\left(2.5,-4,2\right)}^{T}$ m. The initial configuration of the manipulator follows the last column in Table 2.

As we noted before, these uncertainties can be represented in three constant vectors $\Delta a_{z}$, $\Delta a_{\mathrm{zd}}$ and $\Delta a_{\mathrm{zin}}$. Details can be found in the proofs of Properties 1–3.

During the simulations, the vehicle starts at the position (0, 0, 0; 0, 0, 0) (m; rads), and the manipulator starts at the position (0, 0, 0) (rads). The initial point of the feature on the image plane is (216, 230).

In simulations, the controller gains are set as follows:

$$\alpha_{v}=4.5\times 10^{-4},\;K_{s}{=10}^{-3},\;\kappa_{v}=0.01,\;\lambda_{\mathrm{zin}}=0.1,\;\lambda_{\mathrm{zd}}=1\times 10^{-2},\;\lambda_{z}=1\times 10^{-2},\;$$

$$K_{p}=18\times \mathrm{diag}\left\{10,10,10,15,15,15,5,5,5\right\},\;$$

$$W=\left[\begin{array}{cc} 0_{9\times 9} & I_{9}\\ 0_{9\times 9} & 0_{9\times 9} \end{array}\right],\;E=\left[\begin{array}{c} 0_{9\times 9}\\ I_{9} \end{array}\right],\;L=[\begin{array}{cc} I_{9} & 0_{9\times 9} \end{array}],\;p=[\begin{array}{c} 0.1\dot{\zeta }\\ 0.2\dot{\zeta } \end{array}],\;l=[\begin{array}{c} 0.1I_{9}\\ 0.2I_{9} \end{array}],\;{\hat{\gamma }}_{2}(0)=I_{9\times 1}$$

The desired trajectory is given as:

$$\left[\begin{array}{c} u_{d}\\ v_{d} \end{array}\right]=\left[\begin{array}{c} 300+50\sin(0.1t)\\ 200+50\cos(0.1t) \end{array}\right]$$

The proposed scheme is utilized to track the desired circle trajectory in 70 s.

**Figure 5:**
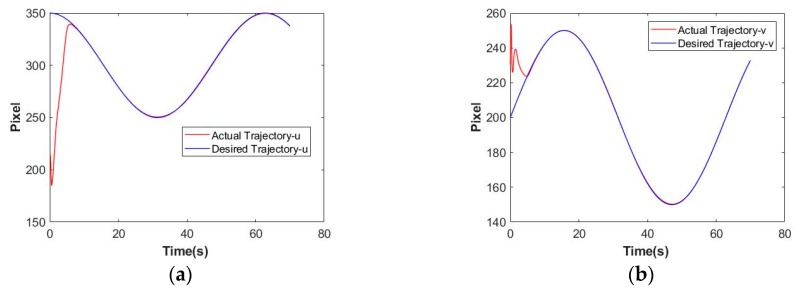

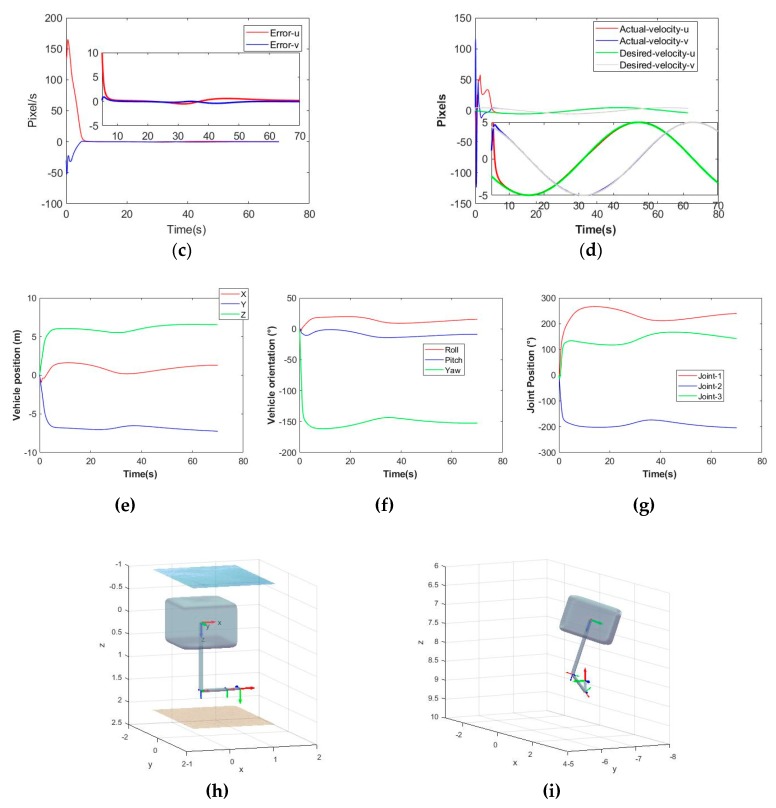
Trajectory tracking performance under the proposed scheme. (**a**) Position tracking performance on the image plane-u; (**b**) Position tracking performance on the image plane-v; (**c**) Position tracking errors on the image plane; (**d**) Velocity tracking performance on the image plane; (**e**) Time histories of vehicle position; (**f**) Time histories of vehicle orientation; (**g**) Time histories of joint position; (**h**) The initial state of the UVMS; (**i**) The final state of the UVMS.

Figure 5a,b show the actual trajectory and the desired trajectory of the feature point. Figure 5c describes the error trajectory during the tracking routine. Figure 5d illustrates the velocity tracking performance under the proposed method. Time histories of vehicle position, vehicle orientation, and manipulator position can be found in Figure 5e–g. The initial state and the final state can be found in Figure 5h,i.

As expectation, rapid convergences have been achieved in both position and velocity tracking performances.

**Figure 6:**
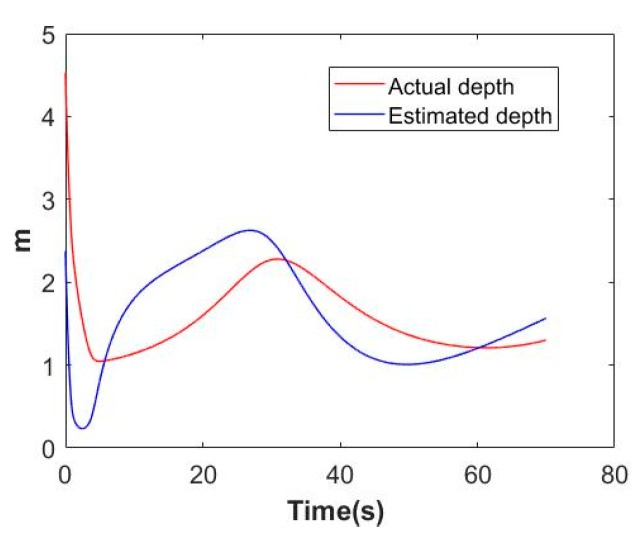
Actual depth and estimated depth of the feature.

**Figure 7:**
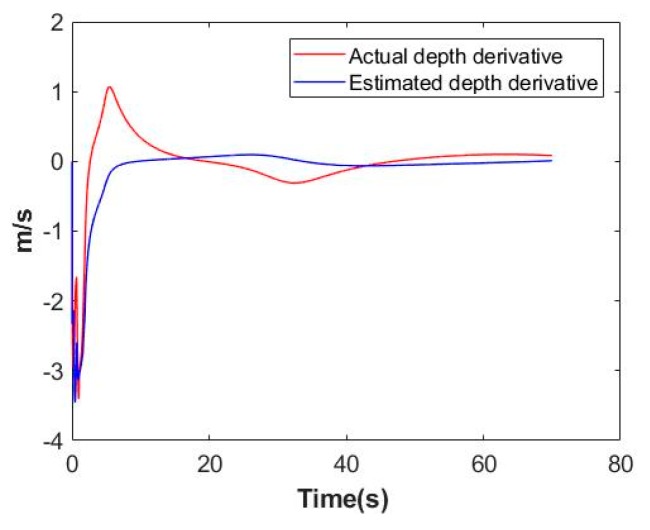
Actual depth derivative and estimated depth derivative of the feature.

From (18) and (21), one can obtain the connection between $a_{\mathrm{zd}}$ and $\dot{z}$, $a_{z}$ and z. Since the dimensions of $a_{z}$ and $a_{\mathrm{zd}}$ are all too large, therefore we choose to display the estimation of feature depth and its derivative to reflect the adaption process, the results can be found in Figure 6 and Figure 7.

From (8), we can define following vector:

$${\left[\alpha ,\beta \right]}^{T}=J_{\mathrm{zin}}(\zeta )\dot{\zeta }=Y_{\mathrm{zin}}(\zeta ,\dot{\zeta })a_{\mathrm{zin}}$$

And its estimation follows:

$${\left[\hat{\alpha },\hat{\beta }\right]}^{T}=Y_{\mathrm{zin}}(\zeta ,\dot{\zeta }){\hat{a}}_{\mathrm{zin}}$$

**Figure 8:**
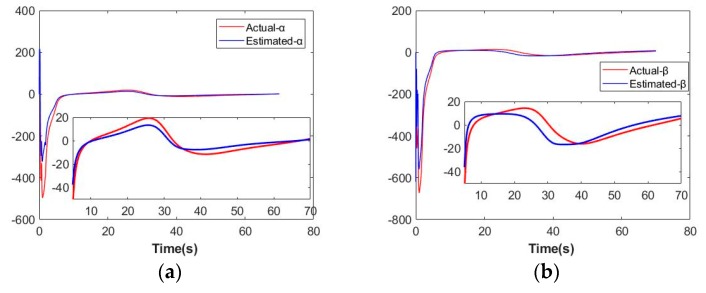
Adaption process on $a_{\mathrm{zin}}$. (**a**) Visual tracking performance on α; (**b**) Visual tracking performance on β.

According to the same reason, we choose to exhibit the actual and estimated values of α and β to illustrate the adaption process of $a_{\mathrm{zin}}$. As Figure 8 illustrates, estimation errors stay bounded.

From stability analysis in previous section, $\Delta a_{z}$, $\Delta a_{\mathrm{zd}}$ and $\Delta a_{\mathrm{zin}}$ are bounded. Therefore, it is reasonable that estimation errors represented in Figure 6, Figure 7 and Figure 8 are bounded.

**Figure 9:**
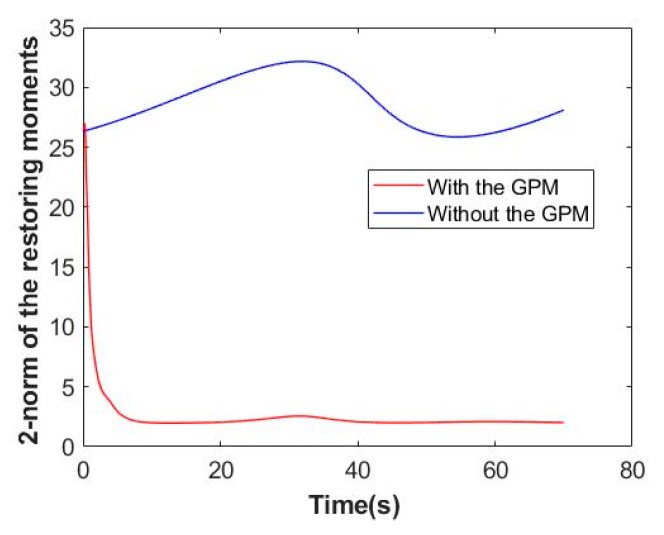
Two-norm of the restoring moments under different reference velocity terms.

Compared with the reference velocity term (33), the proposed method with (34) provide restoring moments optimization by using the GPM. Figure 9 illustrates the two-norm of restoring moments based on (33) and (34), respectively. RMS values of tracking errors based on (33) and (34) can be found in Table 3.

Because the initial error is relatively large, we calculate RMS values from the time point t = 4 s to the end of the simulation. From Table 3, the controller under reference velocity from (33) provides slightly better results in both position tracking and velocity tracking than the one under (34). However, from Figure 9, the results obtained under the GPM from (34) substantially reduce the influence of the restoring moments compared with the one under (33). Considering all these factors, the scheme with the GPM provides better overall performances.

**Figure 10:**
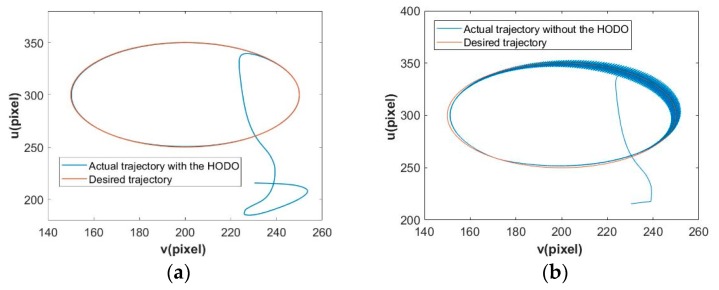
Trajectory tracking performance. (**a**) with the HODO; and, (**b**) without the HODO.

In addition, we compare he tracking performances between the proposed method and the one but without the HODO. As showed in Figure 10, the proposed scheme achieves accurate and smooth results compared with the other one.

**Figure 11:**
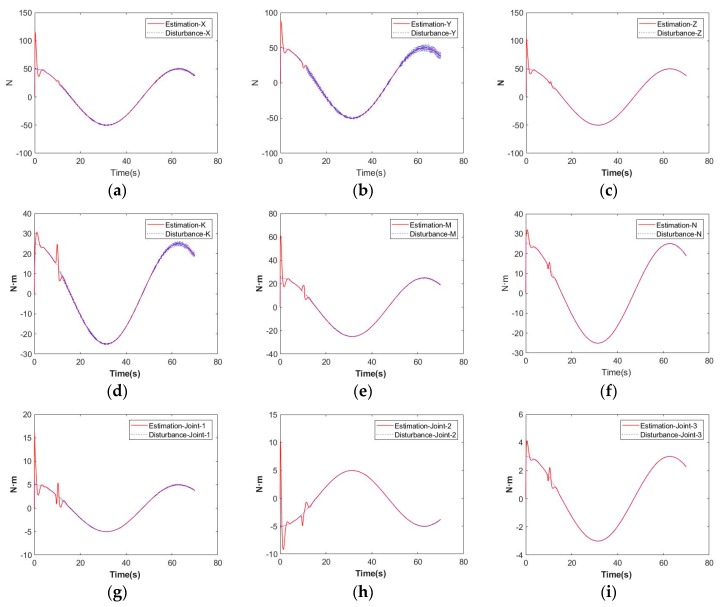
Disturbance estimations on: (**a**) vehicle-X (N); (**b**) vehicle-Y (N); (**c**) vehicle-Z (N); (**d**) vehicle-X (N·m); (**e**) vehicle-Y (N·m); (**f**) vehicle-Z (N·m); (**g**) manipulator-Joint 1 (N·m); (**h**) manipulator-Joint 2 (N·m); (**i**) manipulator-Joint 3 (N·m).

From theoretical analysis in previous section, the HODO achieves BIBO stable, rather than asymptotical stable. But in simulations, the HODO still shows good performances in estimating disturbances in all DOFs. The results can be found in Figure 11. Disturbance estimation error RMS values are given in Table 4.

## 5. Conclusions

In this paper, an uncalibrated visual servoing scheme is proposed for the UVMS with an eye-in-hand configuration camera. Parameters of the vision sensor, kinematics of the UVMS and the target position are supposed to be unknown. Firstly, we introduce a linear separation method to collect kinematics uncertainties into vectors, and this method can be utilized in other free-floating based articulated manipulators. Then, to deal with these kinematics uncertainties, novel adaptive laws are proposed to estimate these vectors, the GPM is utilized to optimize the restoring moments of the system. Moreover, a high order disturbance observer is presented to estimate and compensate lumped disturbances. A novel composite controller is addressed in order to realize the convergence of image errors. The stability of the closed-loop system is proved by utilizing Lyapunov theory. Finally, trajectory tracking simulations based on a 9 -DOFs UVMS are carried out to test the proposed scheme under uncertainties and disturbances.

## Figures and Tables

**Table 1:** Main dynamic parameters of the system.

Item	Vehicle	Link1	Link2	Link3
M(kg)	82.131	2.603	3.520	3.159
$I_{\mathrm{xx}}\cdot (\mathrm{kg}\cdot m^{2})$	4.949	0.0262	0	0
$I_{\mathrm{yy}}\cdot (\mathrm{kg}\cdot m^{2})$	7.362	0.0262	0.1636	0.1636
$I_{\mathrm{zz}}\cdot (\mathrm{kg}\cdot m^{2})$	8.658	0	0.1636	0.1636

**Table 2:** DH parameters of the manipulator.

Joints	$a_{i}/m$	$\alpha_{i}/\mathrm{rads}$	$d_{i}/m$	$q_{i}/\mathrm{rads}\;$	${\hat{d}}_{i}\left(0\right)/m$
**1**	0	π/2	0	$q_{1}$	0
**2**	0	0	0.6	$q_{2}$	0.5
**3**	0	0	0.3	$q_{3}$	0.5

**Table 3:** Tracking error RMS values.

RMS Error	Position Tracking Error	Velocity Tracking Error
With the GPM	2.2198	2.8284
Without the GPM	1.9489	2.4959

**Table 4:** Disturbance estimation error RMS values.

RMS Error	X	Y	Z	K	M	N	Joint-1	Joint-2	Joint-3
Disturbance estimation error	4.9847	3.8484	3.7432	1.3211	2.7874	0.6902	0.7359	1.0303	0.1255
